# High Expression Levels of Total IGF-1R and Sensitivity of NSCLC Cells In Vitro to an Anti-IGF-1R Antibody (R1507)

**DOI:** 10.1371/journal.pone.0007273

**Published:** 2009-10-06

**Authors:** Yixuan Gong, Evelyn Yao, Ronglai Shen, Aviva Goel, Maria Arcila, Julie Teruya-Feldstein, Maureen F. Zakowski, Stanley Frankel, Martin Peifer, Roman K. Thomas, Marc Ladanyi, William Pao

**Affiliations:** 1 Pao Laboratory, Human Oncology and Pathogenesis Program, Memorial Sloan-Kettering Cancer Center, New York, New York, United States of America; 2 Department of Epidemiology and Biostatistics, Memorial Sloan-Kettering Cancer Center, New York, New York, United States of America; 3 Ladanyi Laboratory, Human Oncology and Pathogenesis Program, Memorial Sloan-Kettering Cancer Center, New York, New York, United States of America; 4 Department of Pathology, Memorial Sloan-Kettering Cancer Center, New York, New York, United States of America; 5 Thoracic Oncology Service, Department of Medicine, Memorial Sloan-Kettering Cancer Center, New York, New York, United States of America; 6 Medical Sciences, Oncology, Hoffmann-La Roche Inc., Nutley, New Jersey, United States of America; 7 Max Planck Institute for Neurological Research with Klaus-Joachim Zülch Laboratories of the Max-Planck-Society and the Medical Faculty of the University of Köln, Köln, Germany; 8 Department I of Internal Medicine and Center of Integrated Oncology, University of Köln, Köln, Germany; 9 Chemical Genomics Center of the Max-Planck-Society, Dortmund, Germany; Roswell Park Cancer Institute, United States of America

## Abstract

**Background:**

The IGF receptor type 1 (IGF-1R) pathway is frequently deregulated in human tumors and has become a target of interest for anti-cancer therapy.

**Methodology/Principal Findings:**

We used a panel of 22 non-small cell lung cancer (NSCLC) cell lines to investigate predictive biomarkers of response to R1507, a fully-humanized anti-IGF-1R monoclonal antibody (Ab; Roche). 5 lines were moderately sensitive (25–50% growth inhibition) to R1507 alone. While levels of phospho-IGF-1R did not correlate with drug sensitivity, 4 out of 5 sensitive lines displayed high levels of total IGF-1R versus 1 out of 17 resistant lines (p = 0.003, Fisher's Exact). Sensitive lines also harbored higher copy numbers of *IGF-1R* as assessed by independent SNP array analysis. Addition of erlotinib or paclitaxel to R1507 led to further growth inhibition in sensitive but not resistant lines. In one *EGFR* mutant lung adenocarcinoma cell line (11–18), R1507 and erlotinib co-treatment induced apoptosis, whereas treatment with either drug alone induced only cell cycle arrest. Apoptosis was mediated, in part, by the survival-related AKT pathway. Additionally, immunohistochemical (IHC) staining of total IGF-1R with an anti-total IGF-1R Ab (G11;Ventana) was performed on tissue microarrays (TMAs) containing 270 independent NSCLC tumor samples. Staining intensity was scored on a scale of 0 to 3+. 39.3% of tumors showed medium to high IGF-1R IHC staining (scores of 2+ or 3+, respectively), while 16.7% had scores of 3+.

**Conclusions/Significance:**

In NSCLC cell lines, high levels of total IGF-1R are associated with moderate sensitivity to R1507. These results suggest a possible enrichment strategy for clinical trials with anti-IGF-1R therapy.

## Introduction

Lung cancer is the leading cause of cancer-related death in the United States and worldwide [1]. Lung cancers are currently classified into two major groups depending on the morphologic pathological appearance: small cell lung cancer (SCLC) and non-small cell lung cancer (NSCLC) [2].The latter is comprised of three different subtypes: adenocarcinoma, squamous cell carcinoma, and large cell carcinoma. Despite recent advances in treatments for the disease, the overall 5-year survival in the United States remains only 15% [3], highlighting the need for novel treatment strategies.

One emerging approach involves targeting of the type I insulin-like growth factor receptor (IGF-1R) pathway [4], [5], [6]. This axis plays an important role in mammalian cell growth and development [7]. The main components include the IGF-1R and its highly structurally conserved family member, the insulin receptor (IR). Both receptors consist of two half-receptors, each comprising one extracellular alpha-subunit and one transmembrane beta-subunit that possess tyrosine kinase activity [8]. IGF-1Rs and IRs can homodimerize or form IGF-1R/IR heterodimers. While the IR is activated by insulin, IGF-1R is activated by its ligands, IGF-1 and IGF-II. The availability of IGF-1R ligands is additionally regulated by at least six high-affinity IGF-binding proteins (IGFBP1-6) which broadly function to inhibit IGF bioactivity.

The IGF-1R pathway appears to play important roles in tumorigenesis, metastasis, and resistance to existing forms of anti-cancer therapy [7], [8]. In lung cancer, elevated plasma levels of IGF-1 have been associated with an increased risk of the disease [9]. Conversely, high plasma levels of IGFBP3 have been associated with reduced risk [10]. High IGF-1R expression is associated with poor survival in surgically treated NSCLC patients [11]. In a recent randomized phase II trial involving patients with stage IIIB, IV, or recurrent, treatment-naïve NSCLC, addition of an anti-IGF-1R antibody (CP-751,871; Pfizer) to standard chemotherapy (paclitaxel and carboplatin) led to a 46% objective response rate versus a 32% rate in patients receiving chemotherapy alone [12]. A phase III trial is underway.

Here, we sought to identify molecular biomarkers in NSCLC that may predict for benefit from anti-IGF-1R directed therapy. Specifically, we analyzed parameters that may be associated with sensitivity of 22 NSCLC lines to R1507 (RO4858696; Roche), a fully humanized IgG1 monoclonal antibody directed against the extracellular portion of IGF-1R. It binds with high selectivity to the extracellular domain of IGF-1R (and not to IR), leading to displacement of IGF-1 binding and loss of protein at the cell surface due to receptor internalization and degradation. After identifying one potential biomarker, we analyzed signaling properties affected by anti-IGF-1R treatment in various cell lines and evaluated expression status of the biomarker in 270 NSCLC patient samples.

## Methods

### Cell culture

The human lung adenocarcinoma cell lines H3255, PC-9 and H1975 were described previously [13]. 11–18 was kindly provided by Koichi Hagiwara. A549, HCC827, H2170, H520, H661, H226, SK-MES-1, SW900, H1703, H460 were obtained from ATCC (Manassas, VA). HCC15, H322, H322M, HCC2450, HCC95, HCC2279, HCC2935, and HCC4006 were kindly provided by Adi Gazdar (University of Texas Southwestern Medical Center, Dallas). Cells except SK-MES-1 and SW900 were maintained in RPMI 1640 (ATCC) supplemented with 10% fetal bovine serum and Pen-Strep Solution (both from Gemini Bio-Products, West Sacramento, CA) in a humidified incubator with 5% CO_2_ at 37°C. SK-MES-1 cells were grown in Eagle's Minimum Essential medium (ATCC) supplemented with 10% fetal bovine serum and Pen-Strep Solution. SW900 cells were cultured in Leibovitz's L-15 Medium (ATCC) supplemented with 10% fetal bovine serum and Pen-Strep Solution (both from Gemini Bio-Products, West Sacramento, CA).

### Reagents

Anti-IGF-1R antibody R1507 was kindly provided by Hoffmann-La Roche Inc (Nutley, NJ). Erlotinib was synthesized by the Organic Synthesis Core Facility at Memorial Sloan-Kettering Cancer Center. Paclitaxel was purchased from EMD Biosciences (La Jolla, CA). The Vybrant® Apoptosis Assay Kit #2 was from Invitrogen (Carlsbad, CA). The phospho-receptor tyrosine kinase (RTK) array kit, human IGF-1R ELISA kit, human phospho-IGF-1R ELISA kit, and recombinant human IGF-1 were from R&D Systems (Minneapolis, MN). Recombinant human EGF was from Cell Signaling Technology (Danvers, MA).

### Antibodies

Anti- pERK(Thr202/Tyr204), -ERK, -pAKT (Ser473), -AKT, -pEGFR (Tyr1092), -pIGF-1Rβ (Tyr 1135/1136) and -IGF-1Rβ antibodies, and HRP-conjugated secondary antibodies were purchased from Cell Signaling Technology (Danvers, MA). (Note that EGFR has two numbering systems. The first denotes the initiating methionine in the signal sequence as amino acid −24. The second denotes the methionine as amino acid +1. Commercial antibodies, such as the Y1068-specific anti-phospho-EGFR, use the first nomenclature. In the second nomenclature, which we use here, Y1068 is Y1092.) Anti-total EGFR antibodies were from Santa Cruz Biotechnology (Santa Cruz, CA).

### Growth inhibition and apoptosis assays

Cells were seeded in 96-well plates at a density of 5,000 cells in triplicate and treated with different concentrations of drugs on the following day. The growth status of drug-treated cells was measured at 72 hours post treatment using CellTiter Blue Reagent (Promega, Madison, WI). Annexin V/PI apoptosis assays (Invitrogen) were performed according to manufacturer's instructions.

### Immunoblotting

Cells were scraped from 10 cm petri dishes, washed twice with PBS, and then incubated in RIPA lysis buffer containing protease inhibitor cocktail (Roche Diagnostics, Indianapolis, IN), 40 mM sodium fluoride and 1 mM sodium orthovanadate for 30 min. The supernatants were subjected to SDS-polyacrylamide gel electrophoresis (Invitrogen) followed by blotting with indicated antibodies. Signals were detected by Supersignal® West Pico Luminol/Enhancer Solution (Pierce Biotechnology, Rockford, IL).

### ELISAs

Phospho- and total IGF-1R ELISAs were performed according to manufacturer's instructions (R&D Systems). For phospho-IGF-1R ELISAs, 100 µg of total protein was used for each cell line. For total IGF-1R ELISAs, 25 µg of total protein was used for most of the cell lines. For cells lines with high IGF-1R expression (A549, H322, H322M, 11–18, HCC95), only 5 µg of total protein was used.

### Phospho-RTK array

Phospho-RTK array was performed according to manufacturer's instructions (R&D Systems). 300 µg of total protein was used for each membrane.

### Gene silencing by siRNA

11–18 cells were seed into 6-well plates at a density of 1.25×10^5^ cells/well. 24 hour later, cells were transfected with siRNAs against *GFP* (High Throughput Screening Core Facility, Memorial Sloan-Kettering Cancer Center), human *IGF-1R* or human *GAPDH* (ON-TARGET*plus SMARTpool*, Dharmacon Inc., Chicago, IL) using DharmaFECT 1 transfection reagent as per manufacturer's instruction. Cells were harvested 48 hours after transfection to analyze the level of protein expression by immunoblotting analysis.

### Immunohistochemistry (IHC)

Tissue microarrays (TMAs) were constructed using a fully automated Beecher Instrument, ATA-27, with triplicate cores for each case. Use of human tissues was approved with an institutional waiver and by the human bioutilization committee. The study cohort was comprised of NSCLCs consecutively ascertained at the Memorial Sloan-Kettering Cancer Center (MSKCC) between 1999 and 2006. All biopsies were evaluated at MSKCC, and the histologic diagnosis was based on hematoxylin-eosin staining. TMAs were stained as per manufacturer's instructions on the Ventana Benchmark XT with the anti-IGF-1R rabbit monoclonal antibody (G11; Ventana-Roche, Tuscon, AZ) directed against the C-terminus of the beta chain. Images were obtained with the Olympus DP20 Camera (Center Valley, PA) and taken with the 40X/0.75 objective. Image acquisition and processing software was performed using Adobe Photoshop 7.0 and DP20 software. Cores were scored as follows: 0, no staining; 1, weak focal staining; 2, moderate staining; 3, strong staining with at least 10% of the core showing complete membranous staining. Cores were assigned 1 score and read by 2 pathologists (JTF and MA).

For IHC of cell lines, 30 million cells per line were fixed in 4% paraformaldehyde for 10 minutes, washed with 70% ethanol, and spun down into pellets. The pellets were kept in 70% ethanol in 4°C until they were paraffin-embedded and then processed as above.

### SNP array

As part of a larger effort to characterize the genomes of NSCLC, some authors (MP and RKT) analyzed 84 NSCLC cell lines for chromosomal gene copy number alterations, gene mutations, transcriptional changes and drug response. The detailed description of this collection was published elsewhere [14]. Copy number analysis was performed using 250 K StyI Affymetrix SNP-arrays; data was analyzed using GenePattern software (www.broad.mit.edu/cancer/software/genepattern/) and R (http://www.r-project.org/). All data is MIAME compliant and the raw data has been deposited in a MIAME compliant database GEO (accession number GSE17247).

### Mutational profiling of IGF-1R

The full-length cDNAs of *IGF-1R* obtained from R1507 sensitive lines (11–18, HCC15, H322, H322 and A549) and select resistant lines (HCC95 and H1975) were sequenced via direct Sanger-based sequencing of PCR fragments amplified with M13 tagged primer pairs **(Table S1)**.

## Results

### NSCLC cell lines display differential sensitivities to single agent R1507

R1507 (RO4858696) is a fully human IgG1 monoclonal antibody directed against the extracellular (alpha chain subunit) portion of the human IGF-1R. To confirm that R1507 binds selectively to human IGF-1R, cell lysates from two cell lines (H3255 and 11–18) were immunoprecipitated with R1507 and then immunoblotted with a commercial antibody that recognizes the beta chain subunit of IGF-1R. Compared to whole cell extracts, immunoprecipitation with R1507 readily enriched detection of IGF-1R in lysates from both lines **(Figure S1)**.

**Figure 1:**
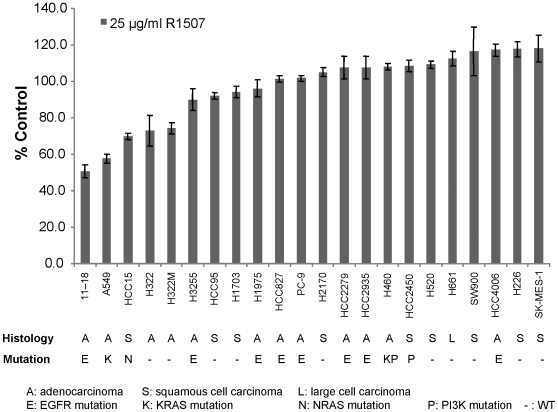
NSCLC cell lines display differential sensitivity to single agent R1507. Various cell lines were treated with R1507 for 72 hours, and cell growth inhibition was measured using CellTiter Blue reagents. The maximum growth inhibition obtained at an R1507 concentration of 25 µg/ml was plotted for each cell line. Data represent the mean ± standard deviation of triplicates.

We next established the sensitivity to R1507 of 22 NSCLC cell lines. These included 12 adenocarcinomas, 9 squamous cell carcinomas and 1 large cell carcinoma, all examined for known *EGFR/KRAS/NRAS/HRAS/PI3K* mutations [15] **(** **Figure 1** **)**. Sensitivity was assessed using a growth inhibition assay that measures a colorimetric signal produced by conversion of resazurin to resorufin, which is directly proportional to the numbers of viable cells. Lines were grown in 10% serum for 72 h in different concentrations (0.1–25 µg/ml) of R1507. None of the lines displayed high sensitivity **(Figure S2)**. Thus, we could not calculate the concentration of drug needed to inhibit growth by 50% (GI_50_) for each line. Instead, we compared the maximum levels of growth inhibition observed across all cell lines that were achieved using an R1507 concentration of 25 µg/ml **(** **Figure 1** **)**. Among the 22 cell lines, 5 (11–18, A549, HCC15, H322, and H322M) displayed 25–50% growth inhibition, whereas the remaining 17 cell lines showed less than 20% inhibition. We chose to define the former lines as “sensitive”, while the latter lines were deemed “resistant”. There was no obvious correlation between R1507 sensitivity and lung cancer histology or mutation status.

### Phospho-IGF-1R levels do not correlate with R1507 sensitivity

To assess whether specific activated receptor tyrosine kinases might correlate with R1507 sensitivity, we incubated lysates from sensitive and resistant lines with a human phospho-RTK array containing antibodies that capture 42 different phosphorylated RTKs. Cell lines were grown in the absence or presence of serum. Lysates from 11–18 cells (the most sensitive line to R1507 in **Figure 1**) contained high levels of phospho-IGF-1R (data not shown). However, none of the remaining four sensitive lines did. We also did not detect any obvious correlation between sensitivity and activated RTKs (data not shown).

**Figure 2:**
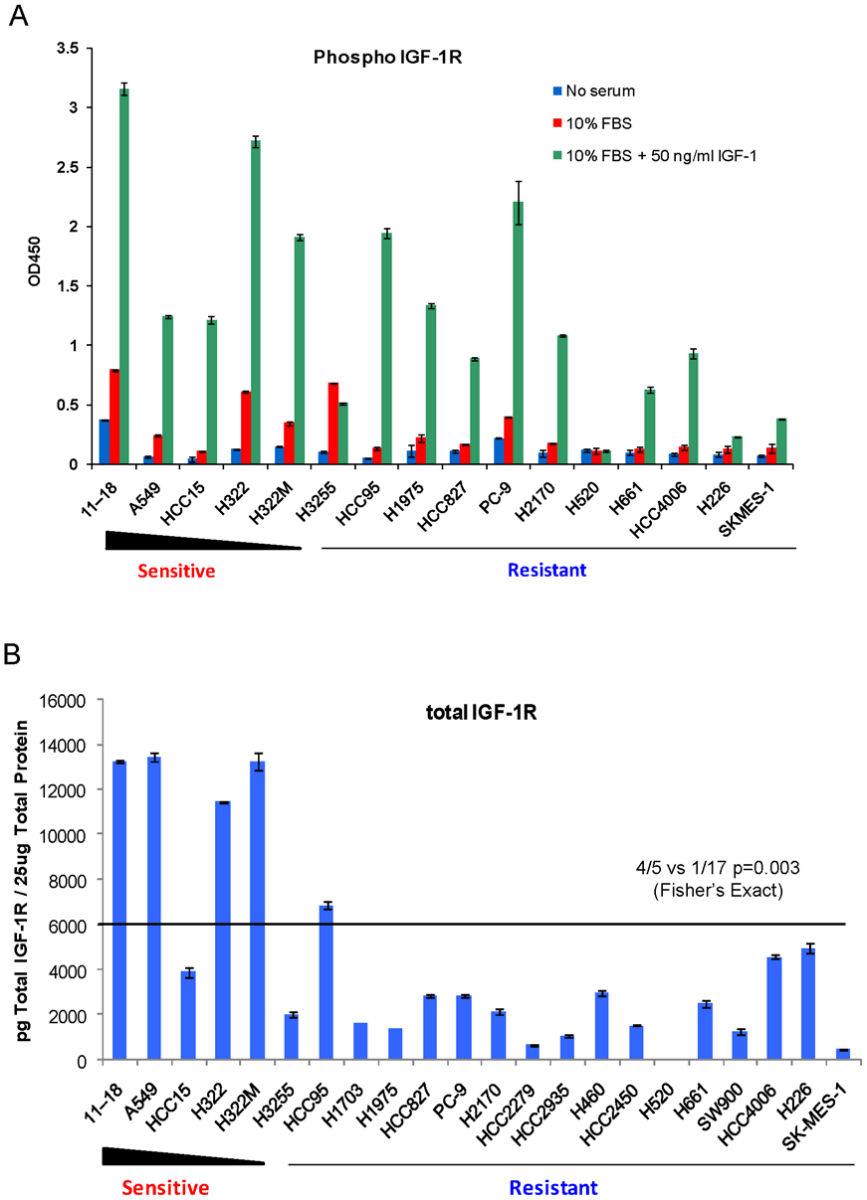
Total IGF-1R levels, but not phospho IGF-1R levels, are associated with R1507 sensitivity in NSCLC cells. A. Cell lines were serum starved overnight, then stimulated with either 10% FBS, or 10% FBS plus 50 ng/ml IGF-1 for 15 minutes. Cell lysates were obtained for phospho IGF-1R ELISA analysis. Y axis is expressed as absorbance at 450 nm, as no standard curve was available to calculate absolute amount. B. Cell lines were serum starved overnight and lysates were obtained for total IGF-1R ELISA analysis. Cell lines are listed according to their relative sensitivity to R1507.

To achieve a more quantitative assessment of the levels of phosphorylated IGF-1R in the various cells, we applied cell lysates from 16 of the 22 lines to a sandwich ELISA assay, designed with an antibody that specifically recognizes IGF-1R and does not cross react with the insulin receptor. Extracts were derived from cells that were serum starved, stimulated with 10% serum, or stimulated with both 10% serum and 50 ng/ml IGF-1 for 15 minutes. Consistent with the results from the phospho-RTK array analysis, levels of phospho-IGF-1R did not correlate with R1507 sensitivity, regardless of cell culture conditions **(** **Figure 2A** **)**.

### Levels of total IGF-1R expression correlate with R1507 sensitivity

We next asked whether expression levels of total IGF-1R correlated with R1507 sensitivity in NSCLC cells. Protein levels were measured quantitatively by ELISA using an antibody specific for both unphosphorylated and phosphorylated forms of the protein **(** **Figure 2B** **)**. Using a cutoff of 6000 picograms per 25 micrograms of total protein, 4 out of 5 sensitive cell lines displayed high total IGF-1R levels, compared to 1 out of 17 resistant lines. This difference was statistically significant (p = 0.003, Fisher's Exact test).

**Figure 3:**
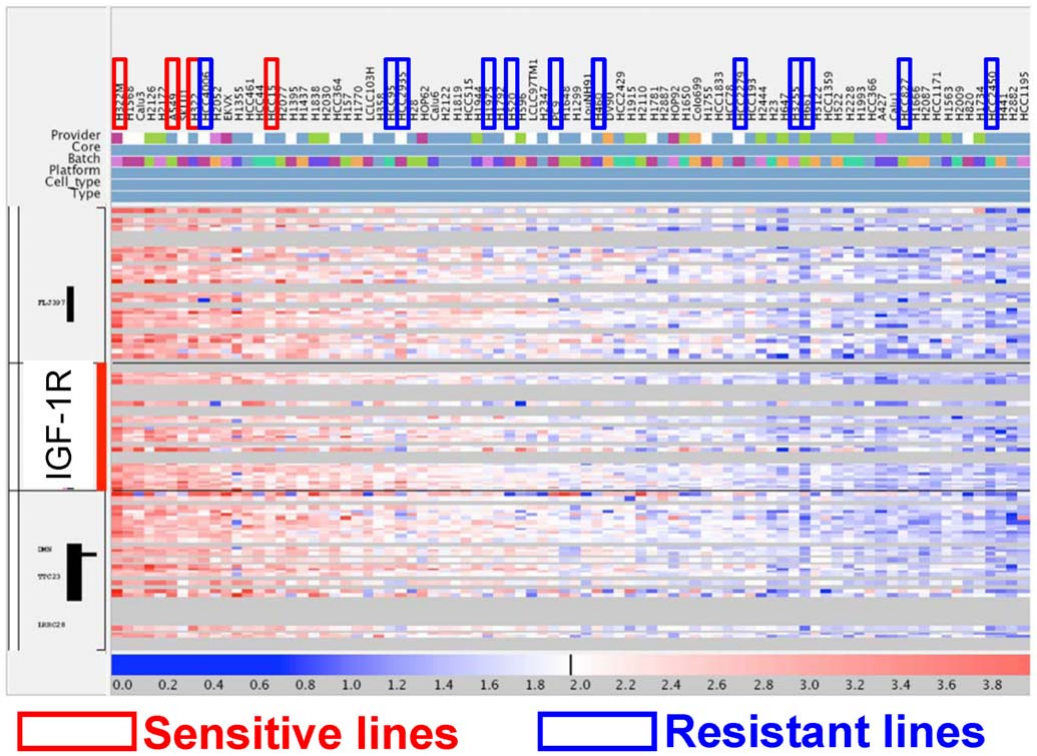
SNP array analysis of *IGF-1R* gene copy number status in a panel of NSCLC cell lines. Cell lines are listed from left to right according to *IGF-1R* gene copy number, highest (red) to lowest (blue). See methods for details.

We also interrogated *IGF-1R* gene copy number status in a panel of NSCLC cell lines that were previously examined using Affymetrix 250K SNP arrays. 4 and 12 of our sensitive and resistant lines, respectively, happened to be included in the SNP array analysis that was performed as part of a separate study [14]. Consistent with the data obtained from the ELISA experiments, cell lines with copy number gain of *IGF-1R* were significantly enriched for R1507-sensitive cell lines **(p = 0.002, Wilcoxon rank sum test;** **Figure 3** **)**. We did not perform additional in situ hybridization to confirm these findings. Notably, none of the 5 sensitive lines harbored any mutations in *IGF-1R* (data not shown).

### IGF-1R signaling is necessary and sufficient for the maintenance of AKT activation in sensitive cells

**Figure 4:**
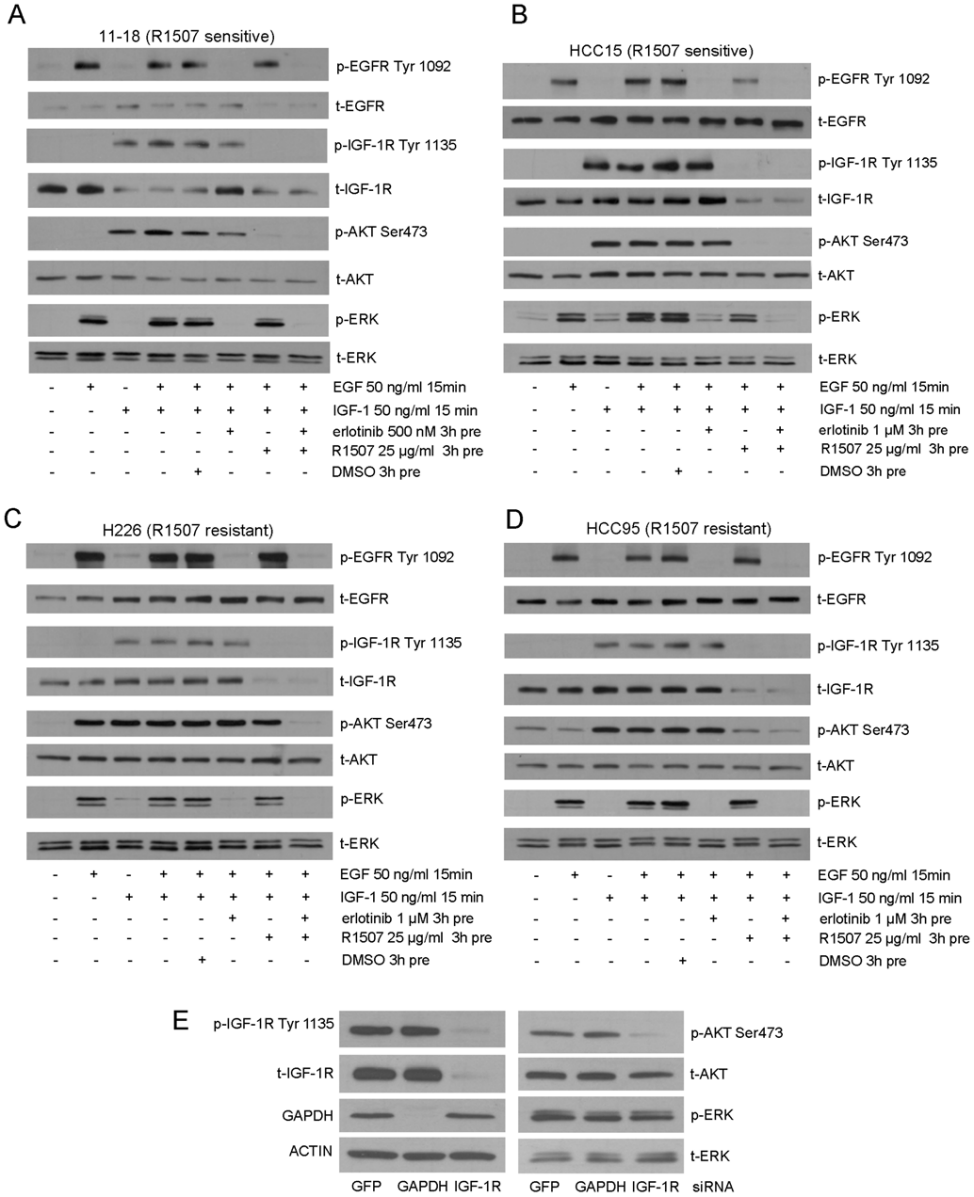
IGF-1R signaling is necessary and sufficient for the maintenance of AKT activation in sensitive cells. A–D. Cells were serum-starved overnight and then stimulated with the indicated concentrations of EGF, IGF-1, or both for 15 minutes. Additional cells were pre-treated with the indicated concentrations of erlotinib, R1507, or both for 3 hours. Cell lysates were analyzed by immunoblotting, using the indicated antibodies. t: total protein; p: phospho-protein. E. 11–18 cells were transfected with siRNAs against the various indicated targets. 48 hours later, cells were harvested, and lysates were analyzed by immunoblotting, using the indicated antibodies.

11–18 cells displayed the most sensitivity to R1507 **(** **Figure 1** **)**. Because they also harbor the L858R mutation of EGFR associated with sensitivity to EGFR tyrosine kinase inhibitors like erlotinib [16], we next investigated interactions between IGF-1R and EGFR in the cells treated under various conditions. Cells were first serum-starved overnight and then stimulated by addition of EGF, IGF-1, or both. Additional cells were stimulated by both EGF and IGF-1 but pre-treated with erlotinib, R1507, or both. As expected, addition of EGF and IGF-1 induced robust phosphorylation of EGFR and IGF-1R, respectively **(** **Figure 4A–D** **)**. Pre-treatment of cells with erlotinib abolished EGFR phosphorylation, while pre-treatment with R1507 abolished phosphorylation of IGF-1R. The latter was accompanied by degradation of total IGF-1R.

Ligand binding to IGF-1R and EGFR initiates a series of phosphorylation events that can lead to activation of both the MAPK pathway involved in cell proliferation and the PI3K/AKT signaling pathway involved in cell survival [7]. Interestingly, in 11–18 cells, AKT was activated by IGF-1 but not EGF stimulation. Conversely, pre-treatment with R1507 but not erlotinib abolished AKT phosphorylation **(** **Figure 4A** **)**. These data indicate that the IGF-1R pathway is both necessary and sufficient for AKT activation in 11–18 cells. Similar results were obtained with extracts from R1507-sensitive HCC15 cells treated in an analogous manner **(** **Figure 4B** **)**.

By contrast, R1507-resistant cell lines displayed different signaling properties. For example, in H226 cells treated in an analogous manner, both EGF and IGF-1 activated AKT, and suppression of the IGF-1R pathway by R1507 alone was not sufficient to eliminate AKT phosphorylation **(** **Figure 4C** **)**. In HCC95 cells, AKT remained phosphorylated despite serum starvation, and R1507 treatment could not abolish AKT activation **(** **Figure 4D** **)**. Similar results were obtained in R1507-resistant PC-9 cells (data not shown). Collectively, these data demonstrate that in R1507-sensitive cells, AKT is solely dependent on IGF-1R for activation, whereas in R1507-resistant cells, AKT can be activated by other pathways or remain constitutively activated.

To verify further that AKT was required for IGF-1R signaling in 11–18 cells, we examined the effect on AKT phosphorylation of knocking down IGF-1R protein expression using gene-specific short interfering RNAs (siRNAs) **(** **Figure 4E** **)**. Consistent with results obtained using R1507 treatment, IGF-1R siRNAs dramatically decreased AKT phosphorylation without affecting ERK phosphorylation in 11–18 cells. Cells treated with control siRNAs against GAPDH did not display these changes.

### Effects of R1507 co-treatment with erlotinib or paclitaxel

**Figure 5:**
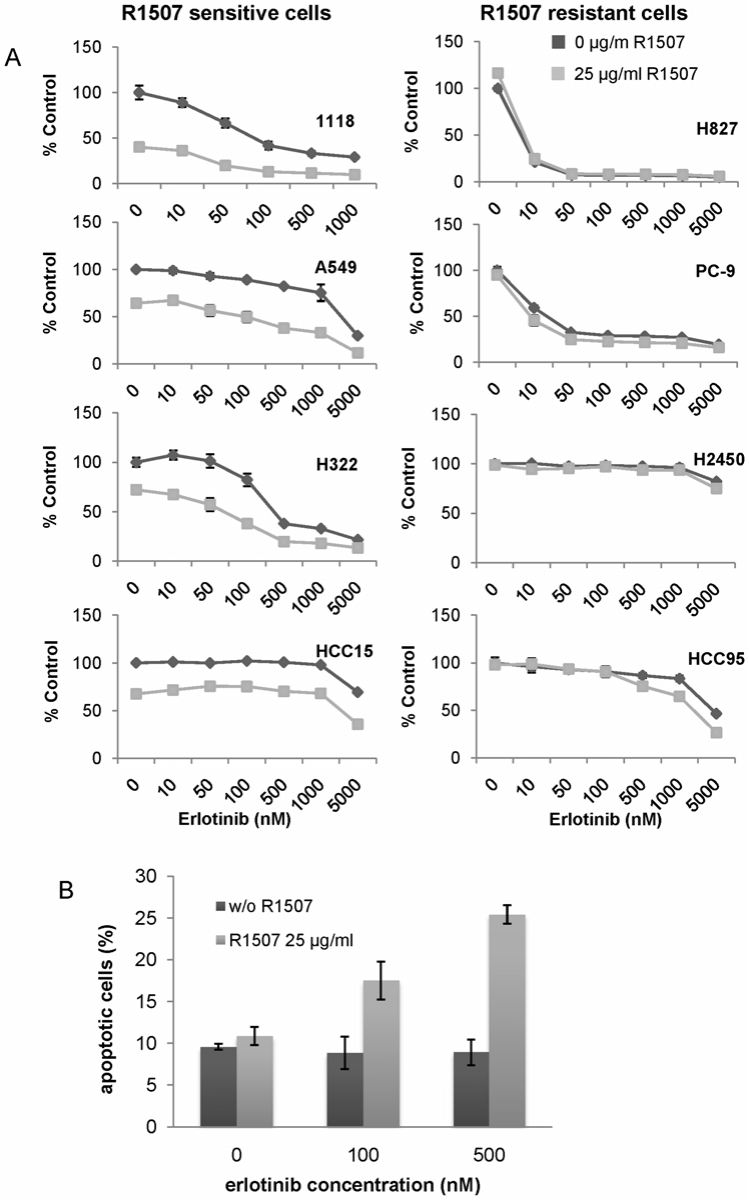
The combinatorial effect of R1507 and erlotinib in NSCLC cell lines. A. R1507 enhances erlotinib-induced growth inhibition in R1507 sensitive cell lines. Various cell lines were treated with increasing concentrations of erlotinib in the absence or presence of 25 µg/ml R1507 for 72 hours and cell growth inhibition was measured by CellTiter Blue reagents. Data represent the mean ± standard deviation of triplicates. B. The combination of R1507 and erlotinib induced apoptosis in 11–18 cells. 11–18 cells were co-treated with 100 or 500 nM erlotinib in the absence or presence of 25 µg/ml R1507 for 24 hours. The percentage of apoptotic cells (annexin V positive cells) was determined by annexin V/PI apoptosis assays.

We examined the effects of adding erlotinib to R1507 on the growth of 11–18 cells **(** **Figure 5A** **)**. Compared to either single agent alone, the combination further inhibited cell growth. Similar results were obtained with the four other R1507 sensitive lines, even though only 11–18 cells harbored a drug-sensitive *EGFR* mutation. By contrast, there was no additive effect in adding erlotinib to R1507 in the lines already resistant to R1507 alone. We were unable to calculate a combination index for the drug combination, as dose response curves derived from treatment of cells with R1507 did not fit into a Hill-type of curve **(Figure S2)**. Analogous results were also obtained by combining R1507 with the chemotherapeutic agent, paclitaxel **(Figure S3)**.

We studied further in 11–18 cells the effect of the combination of R1507 and erlotinib on inducing apoptosis, as measured by standard annexin-V/propidium iodide (PI) assays. Although 11–18 cells harbor a drug-sensitive EGFR L858R mutation, these cells did not die after 24 h of exposure to erlotinib, even at a drug concentration of 500 nanomolar **(** **Figure 5B** **)**. Single agent R1507 at 25 micrograms/milliliter had little effect as well. However, co-treatment with both erlotinib and R1507 induced an increase in annexin-V-positive cells, both at 100 nanomolar (8.9% to 17.5%) and 500 nanomolar (8.9% to 25.4%) erlotinib concentrations (p = 0.007 and 0.0001, two sample t-test). Based upon the analysis of IGF-1R/EGFR signaling **(** **Figure 4** **)**, R1507 contributes to apoptosis induction most likely by eliminating survival-related AKT phosphorylation. Consistent with this notion, we also observed enhanced apoptosis in 11–18 cells using a combination of erlotinib and an experimental PI3K inhibitor (data not shown).

### IGF-1R expression in NSCLC tumors by IHC

**Figure 6:**
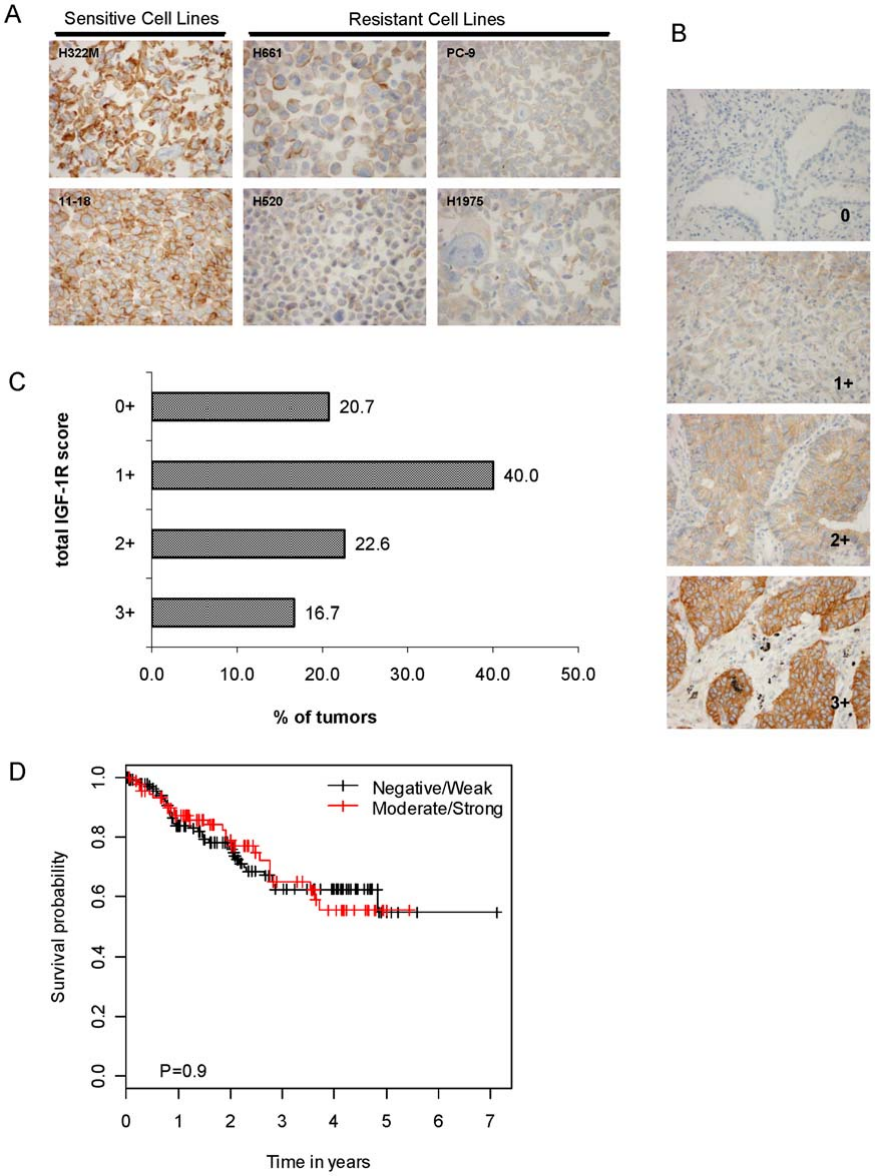
IGF-1R expression in primary NSCLC tissues. A. IGF-1R expression in NSCLC cells lines assessed by IHC analysis. IHC was performed on paraffin-embedded NSCLC cell lines using the total IGF-1R antibody, G11 (see methods). Results were consistent with data obtained from total IGF-1R ELISAs. B. IGF-1R expression in primary NSCLC tumors. IHC staining with G11 was performed on NSCLC tissue microarrays and scored as follows: 0, no staining; 1, weak focal staining; 2, moderate staining; 3, strong staining with at least 10% of the core showing complete membranous staining. Representative images are shown. C. Distribution of scores for total IGF-1R staining in primary NSCLCs. D. Kaplan-Meier curves for overall survival in patients whose tumors scored 0 and 1+ versus 2+ to 3+.

Having identified total IGF-1R protein levels as a potential biomarker of NSCLC cell line sensitivity to R1507, we sought an independent, more clinically applicable assay to assess IGF-1R status in human primary NSCLCs. We obtained a monoclonal antibody (G11) that recognizes the C-terminus of both unphosphorylated and phosphorylated forms of the IGF-1R beta chain. The antibody does not cross react with the insulin receptor (data not shown). We then stained sections made from formalin-fixed NSCLC cell lines and found a striking correlation between IHC staining of the lines and levels of total IGF-1R as determined by ELISAs **(** **Figures 6A** **and** **2B** **)**. Whereas sensitive lines with high levels of total IGF-1R reacted strongly with G11 in a membranous staining pattern, resistant lines with low levels of the protein did not. We next performed IHC analysis on 4 existing tissue microarrays (TMAs) comprised of primary NSCLC tumors from 270 patients. Clinical characteristics of the patients are summarized in **Table 1**. Receptor staining levels varied among tumor samples, from 0 to 3+ (**Figure 6B and 6C** **,** see methods for details). 39.3% of TMA NSCLC tumors showed medium to high IGF-1R staining (scores of 2+ or 3+, respectively), while 16.7% tumors had 3+ scores. IGF-1R expression was mostly localized to tumor cells showing partial to complete strong membranous localization with weaker cytoplasmic localization and tumor cell heterogeneity seen. Surrounding tissue including fibroblasts and inflammatory cells such as lymphocytes and plasma cells were negative, while normal lung alveoli, alveolar macrophages and endothelial cells showed very weak, minimal to negative cytoplasmic staining.

**Table 1:** Association of IGF-1R status with clinical variables.

Variable		IGF-1R(−)	IGF-1R(+)	P
Histology	Adeno	155 (64.6%)	85 (35.4%)	<0.0001
	SCC	5 (20.8%)	19 (79.2%)	
Sex	F	78 (58.6%)	55 (41.4%)	0.58
	M	62 (62.6%)	37 (37.4%)	
Smoking	Never	27 (62.8%)	16 (37.2%)	0.52
	<15 pack/yr	18 (72%)	7 (28%)	
	≥15 pack/yr	93 (59.6%)	63 (10.4%)	
Age (mean)		69 ±10 (n = 140)	68 ±10 (n = 92)	0.25

Adeno – adenocarcinoma. SCC – squamous cell carcinoma. F – female. M – male. Never smoker – smoked less than 100 cigarettes in a lifetime. Age is shown ± standard deviation. Tumors with 2+ to 3+ staining levels were considered IGF-1R(+), while tumors with 0 to 1+ levels were considered IGF-1R(-). Fisher’s exact test was performed to analyze the association of IGF-1R with histology, sex, and smoking history. Two-sample t-test was performed to evaluate IGF-1R association with age.

Note: the numbers do not add up to 270 because not all clinical variables were available for all samples examined.

IGF-1R overexpression was strongly associated with squamous cell carcinoma, as 79.2% of squamous cell lung cancer displayed high IGF-1R staining versus 35.4% of adenocarcinomas (p<0.0001, **Table 1**). There was no significant association between IGF-1R expression levels with gender, smoking history or age. Unlike smoking history or gender in this cohort of patients, IGF-1R expression levels alone were not associated with survival (**Figure 6D** and data not shown).

## Discussion

A variety of strategies to target IGF-1R in cancers are now in preclinical or clinical stages of development [4], [5], [6], [17]. The two main approaches involve anti-IGF-1R monoclonal antibodies and small molecule inhibitors. Currently, there is a limited amount of preclinical data regarding predictors of response to anti-IGF-1R therapy. In this study, through integrated analysis of 22 NSCLC cell lines, we identified high expression levels of total IGF-1R as one potential biomarker of sensitivity to R1507, an anti-IGF-1R antibody. 4 out of 5 sensitive lines displayed high levels of total IGF-1R versus 1 out of 17 resistant lines (p = 0.003, Fisher's Exact). Consistent with this finding, others have recently reported that drug-sensitive sarcomas also have high expression of total IGF-1R [18].

By contrast, we did not find that levels of phospho-IGF-1R were a predictive biomarker for R1507 sensitivity. Both R1507 sensitive cells and resistant cells showed variable levels of phospho-IGF-1R, regardless of culture conditions. This result may be due to the fact that IGF-1R phosphorylation status is a dynamic process which could have varied kinetic properties in different cell lines. We collected samples uniformly 15 mins after ligand stimulation, but peak IGF-1R phosphorylation following administration of IGF could occur at variable times in different cells.

We screened 22 human NSCLC cell lines and identified only 5 lines with moderate response to R1507 single agent treatment in vitro. Surprisingly, these ‘drug-sensitive’ NSCLC cell lines did not undergo more than 25–50% growth inhibition or any significant apoptosis when treated with R1507 alone. We only used one method to assess such sensitivity (i.e. a growth inhibition assay). However, by contrast, the growth of *EGFR* mutant cells that harbor drug-sensitive EGFR kinase domain mutations is nearly completely blocked in this same assay, and apoptosis is induced after analogous treatment with gefitinib or erlotinib [13]. This result suggests that at least among the drug-sensitive NSCLC cells we studied, IGF-1R activity alone is not required for maintenance of the tumor cells in vitro. The receptor does appear to contribute to cell survival, though. For example, 11–18 cells, which harbor an erlotinib-sensitive EGFR mutation (L858R), undergo only G1 arrest in response to erlotinib but undergo apoptosis when co-treated with R1507 and erlotinib.

The lack of significant growth inhibition induced by R1507 in NSCLC cells can be further explained by two additional factors. First, IRs and IGF-1Rs can form hybrid receptors, and it is not yet clear how specific anti-IGF-1R-targeted therapies will interfere with signals emanating from hybrid receptors. However, use of agents that target both IR and IGF-1R was not the focus of this study. Second, we only assessed the effect of R1507 in vitro. R1507 does not elicit antibody-dependent cytotoxicity, but the antibody could still induce more dramatic responses in vivo.

Through analysis of EGFR, IGF-1R, and downstream MAPK and AKT signaling pathways, we found in the R1507-sensitive but not -resistant lines that IGF-1R activation is sufficient and required for AKT activation. Activation of IGF-1R by IGF-1 or inhibition of IGF-1R by R1507 had minimal effect on ERK activation in either R1507 sensitive or resistant lines. Thus, IGF-1R appears to be the major driving force for AKT activation in drug-sensitive cells. The dependence of AKT on IGF-1R signaling was further verified by knockdown of IGF-1R using siRNAs. Consistent with this finding, previous studies have shown in rhabdomyosarcoma cells that phospho-AKT status is controlled predominantly by IGF-1R activation [18]. Insulin receptor substrate 1 (IRS-1) is known to mediate the activation of PI3K by associating with src homology 2 domains of p85 subunit of PI3K upon IGF-1R pathway is activated [19]. In EGFR-dependent cells, the activation of the PI3K/AKT pathway is most likely via ERBB3 [20]. In future studies, it will be interesting to examine the roles of IRS-1 and ERBB3 in mediating PI3K/AKT activation in the R1507-sensitive and -resistant cells.

Through analysis of TMAs with another anti-IGF-1R antibody (G11), we found evidence for high total IGF-1R expression in primary human NSCLCs. 39.3% of tumors had medium to high levels of IGF-1R expression (scores of 2+ to 3+), while 16.7% had high IGF-1R expression (scored as 3+). Higher levels of expression were associated with the squamous cell carcinoma subtype. There was no correlation with gender, smoking history, or overall survival. These results are consistent with a previous study with fewer numbers of patients [21].

In a randomized phase II study of patients with advanced treatment-naïve NSCLC, disease in those with squamous cell carcinoma responded to the combination of paclitaxel, carboplatin and an anti-IGF-1R antibody (CP-751,871) better than those with other histological subtypes [22]. In our IHC studies **(** **Table 1** **)**, a majority of squamous cell lung cancers displayed high levels of IGF-1R expression. These data suggest that squamous cell lung cancers may be more sensitive than lung adenocarcinomas to IGF-1R targeted therapy. However, in our growth inhibition assays, only 1 out of 9 squamous cell carcinoma lines versus 4 out of 11 adenocarcinoma lines displayed moderate sensitivity to R1507. This discrepancy could possibly be explained by phenotypic changes that occur in squamous cell lines when they are grown in vitro. Lung adenocarcinoma cell lines and primary tumors were recently shown to be genetically and transcriptionally similar [14]. However, an analogous analysis has not yet been performed for squamous cell lung cancer cell lines and primary tumors.

In summary, characterization of 22 NSCLC cell lines has lead to identification of high expression levels of total IGF-1R as a predictive marker of relative sensitivity to R1507, an anti-IGF-1R antibody. In such sensitive cells but not resistant cells, additive effects were observed by combining R1507 with other anti-cancer agents. Immunohistochemical analysis of primary NSCLC tumors with an anti-IGF-1R antibody demonstrated that a significant fraction display high levels of the receptor. These results lay groundwork for rationally designing clinical trials to enrich for lung cancer patients that might benefit from treatment with anti-IGF-1R antibodies like R1507.

## Supporting Information

Figure S1 R1507 binds to human IGF-1R. 500 micrograms of total protein from H3255 and 11–18 cell lysates were incubated with 5 µg R1507 overnight followed by 2 hours incubation with protein A/G beads (Santa Cruz Biotechnology). Immunoprecipitates (IP) were separated by SDS/PAGE and then subjected to immunoblotting with a commercial antibody against the beta chain of IGF-1R. WCL - whole cell lysates.
(0.93 MB TIF)

Figure S2 The single agent activity of R1507 in sensitive lines. Various cell lines were treated with R1507 for 72 hours, and growth inhibition was measured using CellTiter Blue reagents.
(0.72 MB TIF)

Figure S3 The combinatorial effect of R1507 and paclitaxel in NSCLC cell lines. R1507 enhances paclitaxel-induced growth inhibition in R1507 sensitive cell lines. Various cell lines were treated with increasing concentrations of paclitaxel in the absence or presence of 25 µg/ml R1507 for 72 hours, and growth inhibition was measured by CellTiter Blue reagents. Data represent the mean ± standard deviation of triplicates.
(0.23 MB TIF)

Table S1 IGF-1R cDNA sequencing primers.
(0.06 MB PDF)
