# Supplementary figures and images for: High Expression Levels of Total IGF-1R and Sensitivity of NSCLC Cells In Vitro to an Anti-IGF-1R Antibody (R1507)
Source: PLoS One. 2009 Oct 6;4(10):e7273. doi: 10.1371/journal.pone.0007273 (PMC2752171; doi:10.1371/journal.pone.0007273)

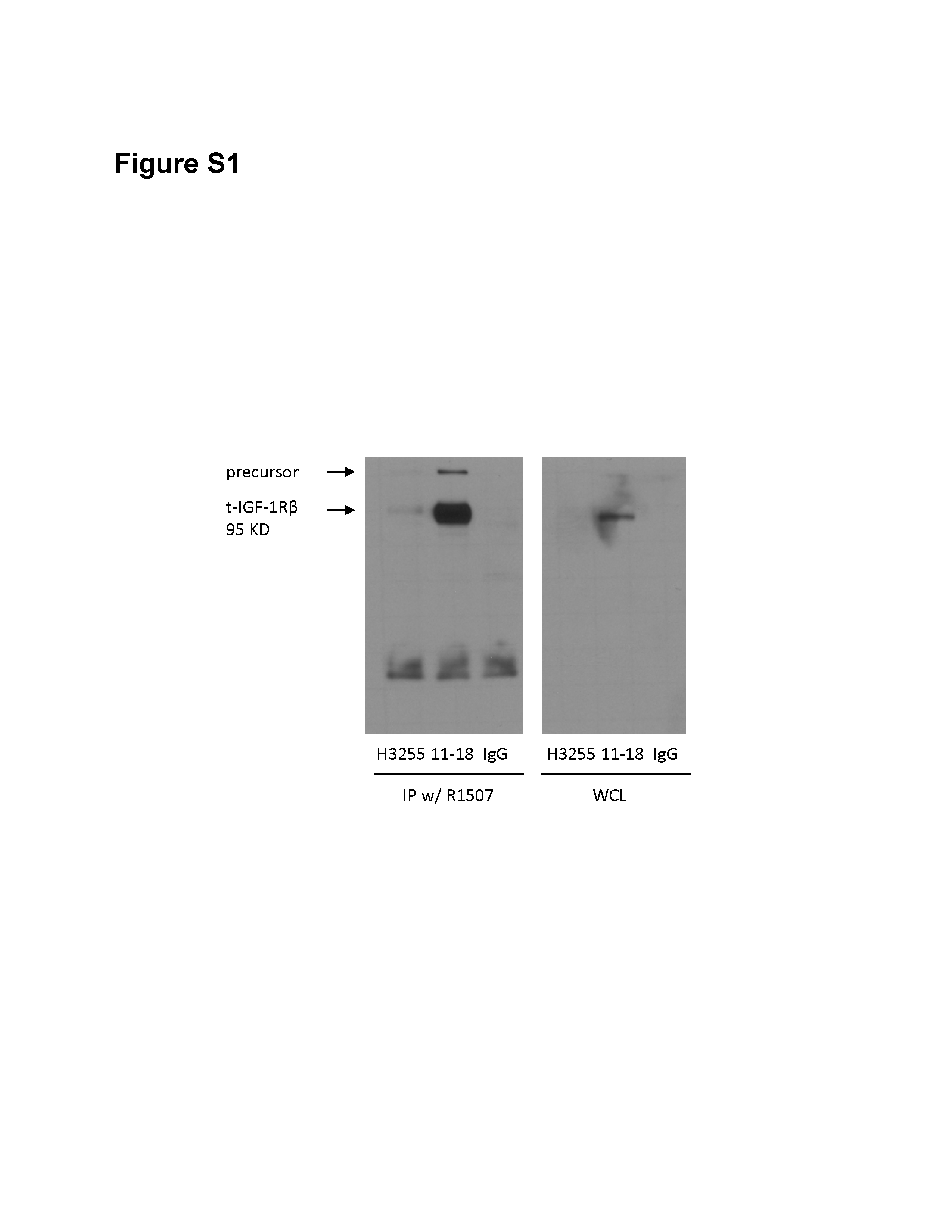

Supplement: Figure S1 — R1507 binds to human IGF-1R. 500 micrograms of total protein from H3255 and 11–18 cell lysates were incubated with 5 µg R1507 overnight followed by 2 hours incubation with protein A/G beads (Santa Cruz Biotechnology). Immunoprecipitates (IP) were separated by SDS/PAGE and then subjected to immunoblotting with a commercial antibody against the beta chain of IGF-1R. WCL - whole cell lysates. (0.93 MB TIF) [file pone.0007273.s001.tif]

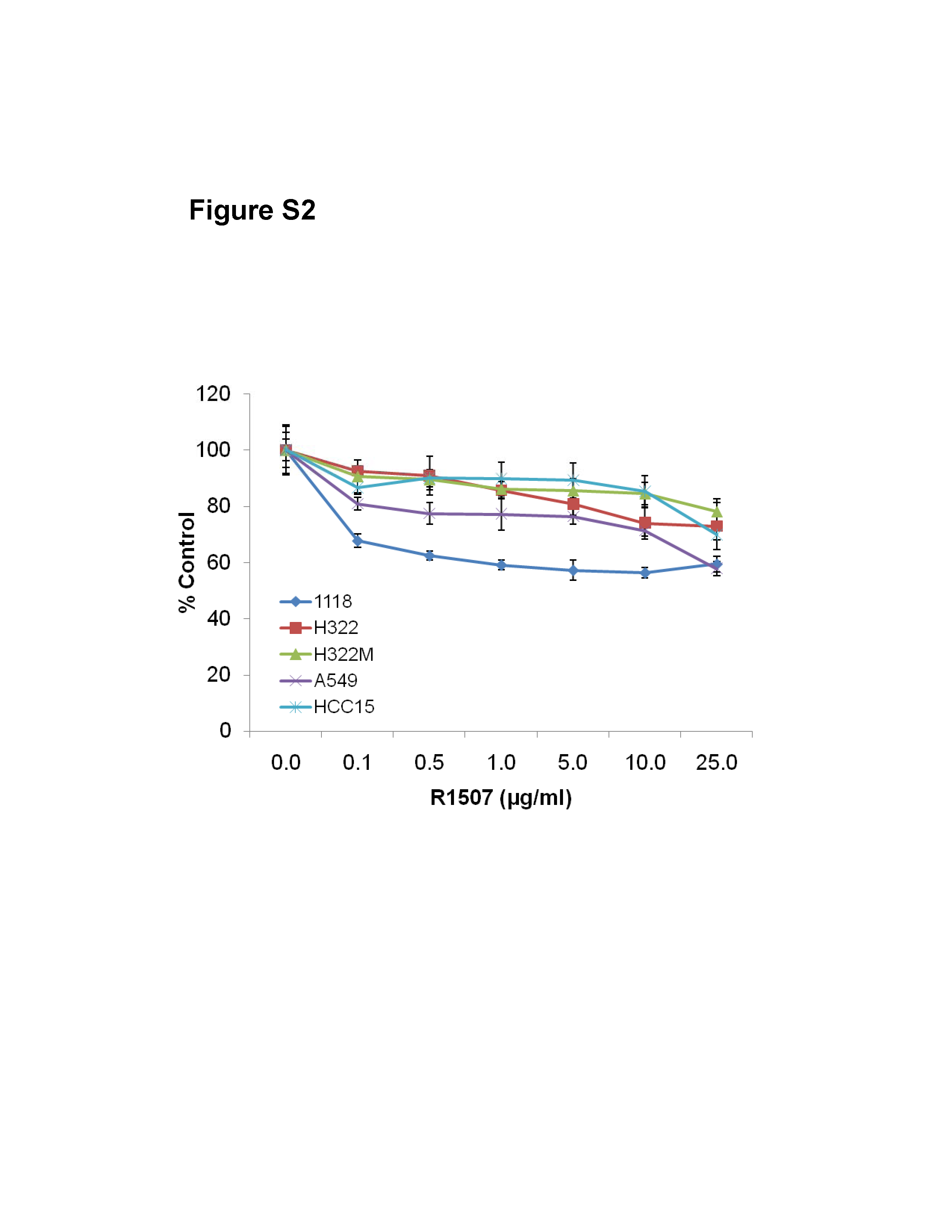

Supplement: Figure S2 — The single agent activity of R1507 in sensitive lines. Various cell lines were treated with R1507 for 72 hours, and growth inhibition was measured using CellTiter Blue reagents. (0.72 MB TIF) [file pone.0007273.s002.tif]

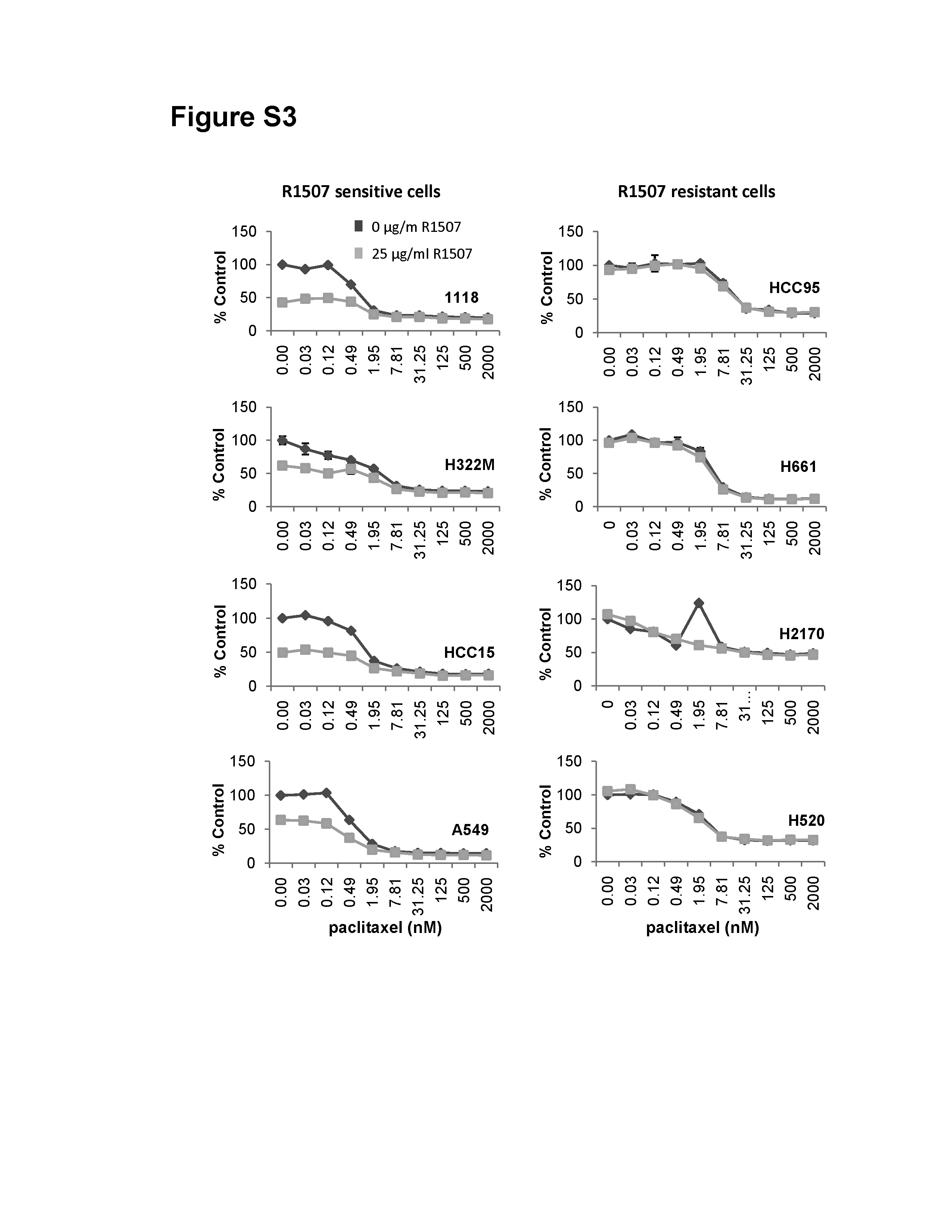

Supplement: Figure S3 — The combinatorial effect of R1507 and paclitaxel in NSCLC cell lines. R1507 enhances paclitaxel-induced growth inhibition in R1507 sensitive cell lines. Various cell lines were treated with increasing concentrations of paclitaxel in the absence or presence of 25 µg/ml R1507 for 72 hours, and growth inhibition was measured by CellTiter Blue reagents. Data represent the mean ± standard deviation of triplicates. (0.23 MB TIF) [file pone.0007273.s003.tif]
