# Supplementary material for: High Expression Levels of Total IGF-1R and Sensitivity of NSCLC Cells In Vitro to an Anti-IGF-1R Antibody (R1507)
Source: PLoS One. 2009 Oct 6;4(10):e7273. doi: 10.1371/journal.pone.0007273 (PMC2752171; doi:10.1371/journal.pone.0007273)
Supplement: Table S1 — IGF-1R cDNA sequencing primers. (0.06 MB PDF) [file pone.0007273.s004.pdf]

Table S1

Primer No.	5’ primer	3’primer
1 (1-502)	GTTTTCCCAGTCACGACATGAAGTCTGGCTCCGGAGG	AACAGCTATGACCATGAGTTATTGGACACCGCATCC
2 (482-982)	GTTTTCCCAGTCACGACGGATGCGGTGTCCAATAACT	AACAGCTATGACCATGAAGGACCTTCACAAGGGATG
3 (961-1460)	GTTTTCCCAGTCACGACTGCATCCCTTGTGAAGGTCC	AACAGCTATGACCATGGAGGCTCTCTCCCCGTTGTT
4 (1441-1945)	GTTTTCCCAGTCACGACAACAACGGGGAGAGAGCCTC	AACAGCTATGACCATGGCCAGCGCACAATGTAGTAA
5 (1921-2420)	GTTTTCCCAGTCACGACCTGAGTTACTACATTGTGCGC	AACAGCTATGACCATGTTGCAGCTGTGGATATCGAT
6 (2401-2901)	GTTTTCCCAGTCACGACATCGATATCCACAGCTGCAA	AACAGCTATGACCATGCAGCCTGCTGTTATTTCTCT
7 (2881-3339)	GTTTTCCCAGTCACGACAAGAGAAATAACAGCAGGCT	AACAGCTATGACCATGAATCATCTTGCTCAGGCTTG
8 (3301-3711)	GTTTTCCCAGTCACGACAATCCAGTCCTAGCACCTCC	AACAGCTATGACCATGACAGTTGTCTGGCTTGTCCA
9 (3661-4110)	GTTTTCCCAGTCACGACGTCCTTCGCTTCGTCATGGA	AACAGCTATGACCATGCAAGGATCAGCAGGTCGAAG
